# Comparing Digital and Fluorescent Imaging for Detecting White Spot Lesions in Orthodontic Patients

**DOI:** 10.1111/ipd.70135

**Published:** 2026-08-17

**Authors:** Prunela Poliçi, Virginia Di Taranto, Antonio Libonati, Giulia Campanella, Gianni Gallusi, Antonio Scarano, Vincenzo Campanella

**Affiliations:** ^1^ Department of Dentistry Catholic University of Our Lady of Good Counsel Our Lady of Good Council University Tirana Albania; ^2^ Department of Clinical Sciences and Translational Medicine Tor Vergata University Rome Italy; ^3^ Department of Innovative Technology in Medicine and Dentistry University of Chieti‐Pescara Chieti Italy

**Keywords:** digital imaging, fluorescent imaging, orthodontic appliances, white spot lesions

## Abstract

**Background:**

To perform orthodontic movements, brackets and ligatures must be placed on the teeth that facilitate plaque accumulation, which can lead to the formation of demineralization lesions.

**Aim:**

The purpose of this comparative study was to detect and measure WSLs using digital and fluorescence‐based methods in patients with malocclusions treated with fixed orthodontic appliances (brackets).

**Design:**

Seventeen patients, age 10–20 years old, 10 girls (58.8%), 7 boys (41.2%), and 273 teeth were analyzed. Their selection was based on six criteria: 1) no vestibular caries or fillings, 2) no dental fluorosis, 3) no hypocalcified teeth, 4) no tetracycline‐stained teeth, 5) no endocrine disorders, and 6) no participation in other experiments.

**Results:**

Digital photography identified a higher number of WSLs than VistaCam Proof at both time points, while agreement between methods was low (Kappa = 0.33 at T0; 0.17 at T1), indicating different lesion detection profiles.

**Conclusions:**

Digital photography and VistaCam Proof showed different WSL detection patterns and low inter‐method agreement. These findings suggest that the two methods are not interchangeable in longitudinal orthodontic monitoring. In the absence of an independent reference standard, the findings should be interpreted in terms of differences in lesion detection and agreement between methods rather than diagnostic performance.

## Why This Paper Is Important to Pediatric Dentists

- To perform orthodontic movements, brackets and ligatures must be placed on the teeth, and they cause areas of demineralization.
- Early diagnosis is essential to prevent progression of cavitated caries.
- Early diagnosis also allows for the adoption of conservative treatments.

## 1 Introduction

One of the main issues associated with the treatment of malocclusions using fixed orthodontic appliances is the development of white spot lesions (WSLs), which represent early manifestations of enamel demineralization.

White spot lesions are opaque areas of enamel that typically appear around orthodontic brackets and represent the earliest sign of demineralization [1]. The presence of brackets and ligatures promotes plaque accumulation, which in turn induces enamel demineralization [2]. After appliance removal, these lesions tend to remineralize; however, the process is slow and may take up to 12 months. This delayed remineralization poses an esthetic concern, particularly in anterior teeth. Keeping WSLs under control, especially in children and adolescents, is important because they can appear as early as the second month of appliance placement [3]. They can cause qualitative and quantitative changes in bacterial plaque, and, if left untreated, can degrade into caries and regeneration may not even be achieved at 100% [4]. Teeth can be lost prematurely. Therefore, healthy treatment of malocclusions requires having good knowledge of the state and dynamics of WSLs before and after the completion of orthodontic treatment. Therefore, diagnostic methods and instruments for WSLs take on particular importance. To measure WSLs, dentists have a large number of measurement methods and instruments at their disposal [4]. They are characterized by specific advantages and limitations, leading to differences in lesion detection rates and detection patterns [5]. According to previous literature, fluorescence‐based methods and visual photographic assessment may differ in their lesion detection characteristics and practical limitations [6, 7]. The digital photography method allows assessment of lesion size and severity. However, its evaluation may be constrained by the Gorelick scoring system, which limits measurement to the cervical third of the tooth, potentially overlooking variations in lesion extent.

The aim of this study was to compare digital photography and fluorescence‐based imaging (VistaCam Proof) in the longitudinal detection of white spot lesions in orthodontic patients and to evaluate the agreement between the two methods before and after treatment. This study obtains WSL data via VistaCam Proof and digital photography. It analyses the collected data to see if the reported values differ or are very similar, and if the values differ, to see if the difference between them is significant or not.

## 2 Materials and Methods

### 2.1 Study Design

The study was conducted at the University from October 2018 to March 2019. It included 17 participants aged 10–20 years (mean age 15 years), 59% girls and 41% boys. Participants gave their consent. For children under 18 years of age, information was provided to their parents. The study protocol was approved by the Ethics Committee of the Catholic University in accordance with the ethical principles of the European Union rules on good clinical practice, according to the declaration of Helsinki [8] and the additional requirements of law. The study compared two methods for the assessment of white spot lesions (WSLs) in orthodontic patients: fluorescence‐based imaging using VistaCam Proof and digital photography assessed with the Gorelick index. WSLs were evaluated at baseline (T0) and at the end of follow‐up (T1), 6 months after bracket placement.

### 2.2 Participants

Participants were selected based on six criteria: no caries or vestibular fillings, no dental fluorosis, no hypocalcified teeth, no tetracycline‐stained teeth, no endocrine disorders, and no participation in another experiment. In the initial phase, 23 candidates were identified. In the final phase, 17 candidates remained after 4 people did not meet the inclusion criteria and 2 people withdrew from the experiment.

This study used a prospective longitudinal parallel‐group design to compare WSL detection by two assessment methods. It analyses data collected from 273 teeth from two groups of patients: (1) one group treated with Duraphat Varnish and (2) the second group not treated with Duraphat Varnish.

### 2.3 Orthodontic Treatment and Fluoride Varnish Protocol

Participants were instructed to maintain good oral hygiene and to brush their teeth at least twice daily with a 1500 ppm F fluoride toothpaste. The experiment went through six steps:

1. The plaque index was measured to all participants;
2. The teeth were polished with a rubber cup and air‐dried;
3. Data on WSL levels before the start of Duraphat Varnish treatment were collected and recorded so that they were ready and available for SPSS data;
4. Brackets were bonded. After the steel brackets were attached, the teeth were immediately isolated with cotton wool rolls and dried with air;
5. Duraphat Varnish was applied following the manufacturer's instructions (Colgate‐Palmolive, New York, NY). Duraphat Varnish applied contained 5% sodium fluoride and was applied only to the experimental group. Patients were instructed not to swallow or rinse their teeth for 5 min and to not eat or drink for 4 h;
6. Data on WSLs levels at the end of treatment were collected 6 months after the use of fixed brackets.

The measurement of the presence and development of WSLs was based on two methods:

1. Fluorescence method;
2. Digital photography method.

Participants were divided into two groups: a fluoride varnish group and a non‐varnish control group. Baseline assessment (T0) was performed before bracket placement and before fluoride varnish application. After bonding of fixed steel brackets, Duraphat Varnish (5% sodium fluoride; Colgate‐Palmolive, New York, NY) was applied only to the fluoride varnish group according to the manufacturer's instructions. Follow‐up assessment (T1) was performed 6 months after bracket placement.

### 2.4 Image Acquisition Procedures

Fluorescence Method consists of software that uses fluorescence to analyze and detect carious lesions and visualize bacterial sites. Fluorescent light stimulates the metabolites of caries‐producing bacteria and makes them glow in red light. Healthy enamel is identified by green, fluorescent light (Table 1). The digital photography method consists of using digital images, analyzed by the naked eye by three experienced dentists who determine the presence and degree of development of WSLs [9]. This method uses the Gorelick scale to determine the degree of demineralization. In analyzing the data, the advantages and disadvantages of both methods were considered (Table 2).

**TABLE 1:** VistaCam proof scoring scale.

Color	Value	Original text (English)
Green	0–1.0	Healthy enamel
Blue	1.0–1.5	Early‐stage enamel caries
Red	1.5–2.0	Deep enamel caries
Orange	2.0–2.5	Dentine caries
Yellow	2.5– > 3.0	Deep dentine caries

*Note:* The colors correspond to the VistaCam Proof classification and indicate the degree of demineralization/caries detected by fluorescence.

**TABLE 2:** Digital imaging scoring scale (Gorelick's).

Category	WSLs presence and level of development
Level 1	None
Level 2	Slight
Level 3	Severe
Level 4	Cavitation

**TABLE 3:** WSL (T_0_).

	Frequency	Percent	Valid percent	Cumulative percent
VistaCam proof				
Green	272	99.6	99.6	99.6
Blue	1	0.4	0.4	100.0
Total	273	100.0	100.0	
Digital images				
No WSL	268	98.2	98.2	98.2
Slight WSL	4	1.5	1.5	99.6
Severe WSL	1	0.4	0.4	100.0
Total	273	100.0	100.0	

*Note:* VistaCam: At T0, almost all teeth are classified as healthy by VistaCam; only one tooth shows early signs of WSL (category “blue”). Digital Images: At T0, the visual assessment of digital images detects five teeth with WSL (4 slight, 1 severe), for an overall prevalence of about 1.8%–1.9%.

To obtain the data, two instruments were used: (1) VistaCam Proof and (2) digital photography. VistaCam is an intraoral camera with a system of three interchangeable heads that produce high resolution images that are visualized and stored digitally. The images are processed by special software. It illustrates the health of the teeth in different colors. Healthy enamel appears translucent. Digital photography is taken inside the mouth. Both instruments have their advantages and disadvantages.

### 2.5 Digital Photography Assessment

The measurement of the presence and degree of development of WSLs is done by the systems specific to each instrument. Table 1 shows the VistaCam scoring scale, while Table 2 shows the scoring scale used by digital photography (Figures 1, 2).

**TABLE 4:** WSL (T_1_).

	Frequency	Percent	Valid percent	Cumulative percent
VistaCam proof				
Valid				
Green	263	96.3	96.3	96.3
Red	4	1.5	1.5	97.8
Orange	5	1.8	1.8	99.6
Yellow	1	0.4	0.4	100.0
Total	273	100.0	100.0	
Digital images				
Valid				
No WSL	241	88.3	88.3	88.3
Slight WSL	29	10.6	10.6	98.9
Severe WSL	3	1.1	1.1	100.0
Total	273	100.0	100.0	

*Note:* Distribution of White Spot Lesions (WSL) at T1 assessed by VistaCam Proof and Digital Images. VistaCam Proof classified 96.3% of teeth as “green” (healthy), with small proportions showing early signs of WSL: “red” (1.5%), “orange” (1.8%), and “yellow” (0.4%). Digital Images identified WSL in 11.7% of teeth (10.6% slight, 1.1% severe), while 88.3% were recorded as having no WSL. These results highlight a higher prevalence and severity of WSL detected by Digital Images compared to VistaCam Proof.

**TABLE 5:** Between‐group and within‐group comparisons (SPSS outputs summarized).

Comparison	Instrument	Test	Statistic(s)	*p* (2‐tailed)	Outcome	Effect size (r)
T0: Treated vs. Untreated	VistaCam Proof	Mann–Whitney U	*U* = 9230; *Z* = −1.041	0.298	No difference between groups at baseline	—
T0: Treated vs. Untreated	Digital Images (Gorelick)	Mann–Whitney U	*U* = 9220.5; *Z* = −0.532	0.595	No difference between groups at baseline	—
T1: Treated vs. Untreated	VistaCam Proof	Mann–Whitney U	*U* = 8591; *Z* = −3.348	0.001	Significant difference (fewer WSL in varnish‐treated group)	—
T1: Treated vs. Untreated	Digital Images (Gorelick)	Mann–Whitney U	*U* = 8546; *Z* = −2.078	0.038	Significant at *α* = 0.05 (trend not significant at *α* = 0.005)	—
Treated group: T0 vs. T1 (paired)	VistaCam Proof	Wilcoxon signed‐rank	*Z* = 0.000	1.000	No change from T0 to T1	0.000
Treated group: T0 vs. T1 (paired)	Digital Images (Gorelick)	Wilcoxon signed‐rank	*Z* = −2.887	0.004	Moderate increase of WSL at T1 vs. T0	0.242
Untreated group: T0 vs. T1 (paired)	VistaCam Proof	Wilcoxon signed‐rank	*Z* = −2.889	0.004	Significant increase of WSL from T0 to T1	0.252
Untreated group: T0 vs. T1 (paired)	Digital Images (Gorelick)	Wilcoxon signed‐rank	*Z* = −4.359	< 0.001	Significant increase of WSL from T0 to T1	0.381

*Note:* Study design: Parallel groups; assessments at baseline (T0) and 6 months (T1). Effect size r computed as |Z|/√N for Wilcoxon tests (paired samples). “Digital Images” refers to visual scoring using the Gorelick scale. Alpha thresholds should be prespecified (e.g., *α* = 0.05); results at T1 differ in significance depending on *α*.

**FIGURE 1:**
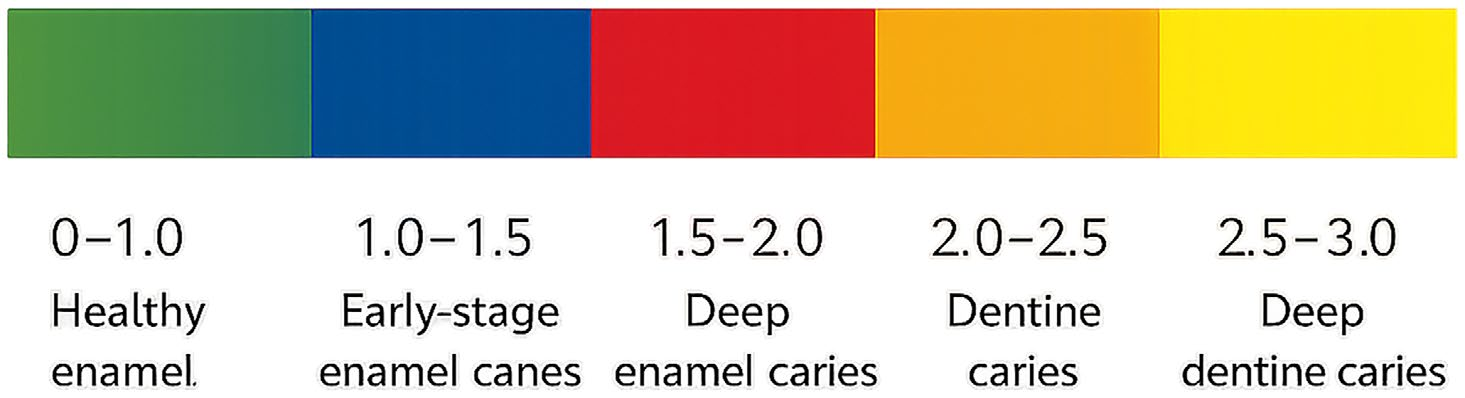
VistaCam Proof Scoring Scale.

**FIGURE 2:**
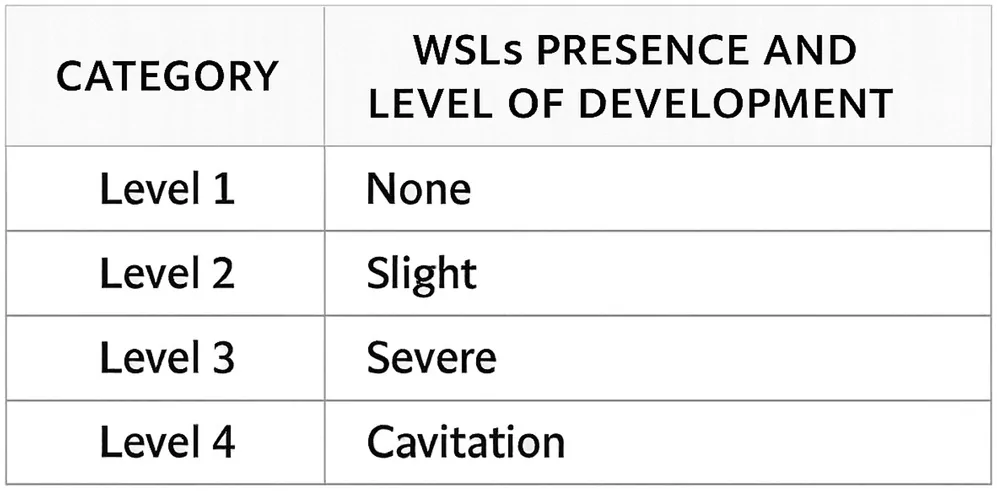
Digital Imaging Scoring Scale (Gorelick's).

This study analyses the lesion detection rates of the instruments by comparing data collected by VistaCam Proof and digital photography about the presence and degree of development of WSLs on the following elements: (1) before and after treatment, (2) changes in WSLs from T_0_ to T_1_, (3) the presence and change of WSLs between the two groups, and (4) the presence and change of WSLs within the same group. By comparing the values reported by VistaCam Proof and digital photography, the purpose of the analysis is to see (1) whether the instruments lead to the same or different conclusions about the presence and extent of WSLs development; (2) whether the conclusions drawn based on the values reported by the instruments are the same or different; and (3) whether the two methods provide similar or different assessments of WSL presence and progression over time. Blinding procedures between the two assessment methods were not formally recorded.

The data were analyzed with SPSS. Parametric and nonparametric statistical techniques were used for data analysis. The confidence level in the results was set at *p* < 0.05.

## 3 Results

VistaCam provides good depth of field and image quality, enables plaque visualization, and allows for early detection of tooth decay. Its images can be analyzed with dedicated software, stored for future reference, and used to aid diagnosis and patient communication. However, as VistaCam has only been in use for a few years, its long‐term reliability is still under evaluation. Digital photography is straightforward to use, can capture all visible stains in the mouth, and helps determine the extent of deterioration. However, its lesion detection rates are limited to the cervical third of the tooth, which is the area most likely to be affected by brackets.

### 3.1 WSL Prevalence at Baseline and Follow‐Up

At baseline, both methods detected WSLs, but digital photography identified a greater number of lesions than VistaCam Proof. At follow‐up, lesion prevalence increased with both methods, although the increase was more pronounced with digital photography. Agreement between methods was low at both time points and decreased further after treatment.

### 3.2 Longitudinal Changes From T0 to T1

From T0 to T1, both methods showed an increase in the number of detected WSLs. The magnitude of this increase was greater for digital photography than for VistaCam Proof, indicating different longitudinal detection patterns between the two methods.

### 3.3 Fluoride Varnish versus Non‐Varnish Control

No clear difference between the fluoride varnish group and the non‐varnish control group was observed at baseline. At follow‐up, subgroup findings differed depending on the assessment method, with VistaCam Proof and digital imaging yielding nonidentical patterns of group comparison (Tables 1, 2, 3, 4, 5, 6).

**TABLE 6:** Agreement between instruments (VistaCam proof vs digital images).

Time point	κ (Kappa)	Approx. Std. error	Approx. t	*p*	Interpretation	*N*
T0	0.330	0.247	7.967	< 0.001	Fair agreement	273
T1	0.170	0.080	3.509	< 0.001	Slight agreement	273

*Note:* Kappa interpretations follow common thresholds (e.g., slight: 0.01–0.20; fair: 0.21–0.40). Lower κ at T1 indicates decreasing interchangeability.

## 4 Discussion

The results of the present study suggest that VistaCam Proof and digital photography are no exception to this challenge. The present study showed that digital photography identified a greater number of WSLs than VistaCam Proof, while agreement between the two methods was low. These differences influence the interpretation of findings and, consequently, the conclusions that can be drawn. The discrepancy between methods may reflect differences in image acquisition, visualization, scoring systems, and lesion classification criteria. However, because no independent reference standard was used, the present study cannot determine whether one method over‐detected or under‐detected true lesions.

Agreement between the two systems ranged from slight to fair, a result consistent with recent studies [10, 11, 12]. This raises the question of what factors might explain such discrepancies. The higher detection rate of digital photography may be attributed to its methodological advantages, as well as limitations of fluorescence‐based methods [13]. Digital photographs capture the entire dentition and enable visualization of lesion extent and progression. In contrast, VistaCam Proof examines only exposed enamel and dentine, with limited ability to detect adjacent or occlusal lesions [14]. The discrepancy between methods may reflect inherent differences in image acquisition, visualization, scoring criteria, and lesion classification systems. However, in the absence of an independent reference standard, the present study cannot determine whether one method over‐detected or under‐detected true lesions. Conversely, VistaCam Proof showed a lower lesion detection rate and has been associated with discrepancies in lesion identification compared with other assessment methods, as previously reported [15, 16]. Differences in diagnostic the lesion detection rates across measurement instruments have long been reported in the dental literature [4, 10, 11, 17, 18, 19, 20, 21, 22]. Although these parameters are commonly used in diagnostic studies, they cannot be formally estimated in the present investigation because no independent reference standard was available. Detection rate reflects the number of lesions identified by a given method. Variations between methods may result in differences in lesion detection and classification, leading to discrepant findings [5, 23]. The literature further reports limited agreement between photographic systems, visual indices (ICDAS‐II, Gorelick), and fluorescence‐based techniques, underlining the importance of examiner calibration and the adoption of standardized protocols to reduce inter‐operator variability [11, 22]. Previous studies have reported discrepancies between visual, photographic, and fluorescence‐based systems for early enamel lesion assessment. In this context, the low agreement observed in the present study is consistent with the broader literature indicating that different WSL assessment systems may not be interchangeable.

Several authors recommend combining different scoring systems, such as ICDAS‐II with Gorelick, to strengthen diagnostic robustness, particularly in multicenter or longitudinal studies [10, 22].

Historically, the intraoral camera was introduced in the 1990s by Gordon Christensen as an adjunctive tool for caries detection, offering magnification (up to 10×) and a broad viewing angle [24]. However, subsequent studies reported limitations in fluorescence‐based devices. For example, Seremidi [16] (2012) found poor performance of VistaCam Proof in detecting early enamel lesions, as it cannot quantify changes in the internal enamel structure. More recently, Salama [15] (2022) reported that the VistaCam HD Smart achieved **a** low detection rate sensitivity, high specificity and an overall accuracy of 67%, again highlighting discrepancies with ICDAS‐II in identifying enamel caries. The lower number of lesions identified by VistaCam Proof compared with digital photography may reflect differences in device characteristics, scoring thresholds, and image interpretation.

The system provides depth information, color‐coded images, early caries detection, plaque visualization, and assessment of demineralization and remineralization dynamics [14]. Nonetheless, differences in detection approaches may result in discrepant findings between methods. Nonetheless, such differences in detection approaches may result in discrepant findings between methods. In contrast, digital photography may be affected by variability in illumination and calibration, as well as by limitations of the evaluation system in the cervical third of the tooth, especially in the presence of orthodontic brackets. These factors may contribute to differences in lesion detection and classification [13]. Emerging diagnostic modalities—including optical coherence tomography (OCT), quantitative microradiography and luminescence‐based systems—have shown promising potential for quantifying and monitoring WSLs, although they are not yet integrated into routine clinical practice [13, 25, 26]. In combination with standardized visual assessment, these techniques may shape the future of orthodontic diagnostics. Furthermore, current evidence suggests that diagnosis should not rely on a single method. A multimodal approach, combining visual evaluation, digital photography, and fluorescence‐based tools, may offer complementary lesion detection and classification profiles, enhancing the monitoring of remineralization strategies [10, 11, 27]. Establishing harmonized protocols and multicenter collaborations is also essential to standardize data collection, facilitate large‐scale studies, and minimize operator‐ and instrument‐related variability [10, 27]. Finally, it should be noted that the present study is limited by its small sample size, short follow‐up, and single‐center design. These factors restrict the generalizability of the findings and highlight the need for multicenter studies with extended observation periods. Identifying the most effective method for assessing WSLs remains challenging [14].

An additional consideration is the natural tendency of post‐orthodontic white spot lesions to undergo partial remineralization over time. This process may affect the visual appearance and fluorescence characteristics of lesions and could contribute to differences in how longitudinal changes are captured by the two methods. In this study, digital photography and VistaCam Proof showed different WSL detection patterns, and the level of agreement between the two methods ranged from fair at baseline to slight after treatment. These findings indicate that neither method should be used in isolation. As previously suggested [5, 28], combining both techniques offers a more reliable diagnostic approach. Consistent with recent reviews, integrated approaches combining standardized indices, digital photography, and fluorescence‐based tools may provide complementary information, improving lesion detection and supporting reliable follow‐up [10, 11, 25]. The implementation of shared protocols and multicenter collaborations will further harmonize data collection and reduce variability related to operator and instrument.

### 4.1 Limitation

A major limitation of this study is the absence of an independent reference standard; therefore, the findings should be interpreted in terms of differences in lesion detection patterns and inter‐method agreement, without implying superiority of one method. An additional limitation of this study is the absence of formal inter‐ and intra‐examiner reliability assessment. Digital photograph scoring using the Gorelick index involves a subjective component, and although images were evaluated by three experienced dentists, no reproducibility analysis was prospectively performed. In addition, fluorescence‐based assessment with VistaCam Proof may also be influenced by operator handling and positioning. Therefore, part of the variability observed between methods may reflect examiner‐ or operator‐related variation rather than only true differences between the two assessment systems.

Digital photography and VistaCam Proof yielded different patterns of WSL detection in orthodontic patients, with digital photography identifying a greater number of lesions and low agreement observed between methods. These findings indicate that the two approaches are not interchangeable for longitudinal monitoring. Without an independent reference standard, the present study does not allow evaluation of diagnostic performance, and interpretations are restricted to observed differences in lesion detection and inter‐method agreement.

Future research should prioritize extended follow‐up periods, larger and more heterogeneous cohorts, and the systematic integration of examiner calibration and emerging imaging technologies. Collaborative multicenter trials will be key to establishing harmonized diagnostic protocols and advancing both research and clinical management of WSLs.

### 4.2 Why This Article Is Important for Pediatric Dentists

Early diagnosis and monitoring of white spot lesions (WSL) are crucial in pediatric and adolescent patients undergoing orthodontic treatment. The use of medical guidance can help pediatric dentists improve early diagnosis, optimize preventive strategies, and reduce the progression of enamel demineralization. In conclusion, a multimodal diagnostic approach can improve clinical decision‐making and long‐term esthetic outcomes in young patients.

## Author Contributions

V.C. designed the research study. V.D.T., A.L., G.C., and G.G., conducted the literature search and performed data extraction. A.S., G.C., and V.C. conceptualized the manuscript and V.D.T., A.L., G.C., G.G., and, A.S., provided the datasets. A.S., and V.C. wrote a draft of the manuscript. All authors interpreted results and revised critically the manuscript. All authors approved final version.

## Funding

The authors have nothing to report.

## Ethics Statement

The study protocol was approved with No. 391 by the Ethics Committee of the Catholic University “Our Lady of Good Counsel” in Tirana, Albania.

## Consent

The authors have nothing to report.

## Conflicts of Interest

The authors declare no conflicts of interest.

## Data Availability

The data that support the findings of this study are available from the corresponding author upon reasonable request.
